# Supplementary material for: Accelerated linear algebra compiler for computationally efficient numerical models: Success and potential area of improvement
Source: PLoS One. 2023 Feb 24;18(2):e0282265. doi: 10.1371/journal.pone.0282265 (PMC9956871; doi:10.1371/journal.pone.0282265)
Supplement: Attachment — Submitted filename: response_to_reviewers.docx [file pone.0282265.s002.docx]

**Accelerated linear algebra compiler for computationally efficient numerical models: success and potential area of improvement**

**Response to reviewers**

Xuzhen He

Journal Requirements:

When submitting your revision, we need you to address these additional requirements.

1. Please ensure that your manuscript meets PLOS ONE's style requirements, including those for file naming. The PLOS ONE style templates can be found at

https://journals.plos.org/plosone/s/file?id=wjVg/PLOSOne_formatting_sample_main_body.pdf and 
https://journals.plos.org/plosone/s/file?id=ba62/PLOSOne_formatting_sample_title_authors_affiliations.pdf

The manuscript has been revised according to the PLOS ONE style requirement. Revisions include (1) changing the round brackets to square brackets in references, (2) changing the font size of the headings, and (3) changing figure references according to the requirements. These formatting revisions are not shown in the marked-up copy of the manuscript to avoid the diffusion of information about technical revisions.

2. Please note that PLOS ONE has specific guidelines on code sharing for submissions in which author-generated code underpins the findings in the manuscript. In these cases, all author-generated code must be made available without restrictions upon publication of the work. Please review our guidelines at https://journals.plos.org/plosone/s/materials-and-software-sharing#loc-sharing-code and ensure that your code is shared in a way that follows best practice and facilitates reproducibility and reuse.

The codes used to reproduce the results of the current study are uploaded onto the GitHub repository and are publicly available at <https://github.com/xuzhen-he/XLA_numerical_models> (DOI: 10.5281/zenodo.7523671)

3. Thank you for stating the following in the Acknowledgments Section of your manuscript:

"This work was supported by the Australian Research Council Discovery Early Career Researcher Award (DECRA; DE220100763)"

We note that you have provided funding information that is not currently declared in your Funding Statement. However, funding information should not appear in the Acknowledgments section or other areas of your manuscript. We will only publish funding information present in the Funding Statement section of the online submission form. Please remove any funding-related text from the manuscript and let us know how you would like to update your Funding Statement. Currently, your Funding Statement reads as follows:

"XH was supported by the Australian Research Council (https://www.arc.gov.au/) Discovery Early Career Researcher Award (DECRA; DE220100763). The funder had no role in study design, data collection and analysis, decision to publish, or preparation of the manuscript."

Please include your amended statements within your cover letter; we will change the online submission form on your behalf.

I have deleted the funding information in the Acknowledgements section, and I do not need to amend the original Funding Statement.

4. In your Data Availability statement, you have not specified where the minimal data set underlying the results described in your manuscript can be found. PLOS defines a study's minimal data set as the underlying data used to reach the conclusions drawn in the manuscript and any additional data required to replicate the reported study findings in their entirety. All PLOS journals require that the minimal data set be made fully available. For more information about our data policy, please see http://journals.plos.org/plosone/s/data-availability.

""Upon re-submitting your revised manuscript, please upload your study’s minimal underlying data set as either Supporting Information files or to a stable, public repository and include the relevant URLs, DOIs, or accession numbers within your revised cover letter. For a list of acceptable repositories, please see http://journals.plos.org/plosone/s/data-availability#loc-recommended-repositories. Any potentially identifying patient information must be fully anonymized.

Important: If there are ethical or legal restrictions to sharing your data publicly, please explain these restrictions in detail. Please see our guidelines for more information on what we consider unacceptable restrictions to publicly sharing data: http://journals.plos.org/plosone/s/data-availability#loc-unacceptable-data-access-restrictions. Note that it is not acceptable for the authors to be the sole named individuals responsible for ensuring data access.

We will update your Data Availability statement to reflect the information you provide in your cover letter.

The data generated and analysed during the current study are compiled into a spreadsheet and uploaded as supporting information to make it publicly available.

5. Please review your reference list to ensure that it is complete and correct. If you have cited papers that have been retracted, please include the rationale for doing so in the manuscript text, or remove these references and replace them with relevant current references. Any changes to the reference list should be mentioned in the rebuttal letter that accompanies your revised manuscript. If you need to cite a retracted article, indicate the article’s retracted status in the References list and also include a citation and full reference for the retraction notice.

The reference list is double-checked. It is complete, and correct and does not cite retracted articles.

Review Comments to the Author

Please use the space provided to explain your answers to the questions above. You may also include additional comments for the author, including concerns about dual publication, research ethics, or publication ethics. (Please upload your review as an attachment if it exceeds 20,000 characters)

Reviewer #1:

1. in the introduction sections, author should clearly mentioned the novelty of the proposed model.

Thank you for this suggestion. The abstract and the introduction are revised to explicitly mention the novelty of this study, which is summarised as: (1) This study is the first of its kind to examine the efficiency of XLA for a general category of numerical models and the efficiency is examined in a stringently way by comparing the performance of numerical models implemented using XLA on a computing platform with the maximum possible performance achieved on that computing platform. (2) The computing speed of element-wise operations and finite-difference models are found faithfully modelled by two simple equations with very few easily-measurable parameters. These two equations suggest methods to roughly estimate the computing speed of numerical models. (3) XLA is found efficient for some numerical models and for some computing platforms but is not for others, and potential areas of improvement are suggested for the non-optimal scenarios.

2. the quality of figures needs more improvement

Thank you. The figure format has been changed from jpeg to tiff to follow the journal requirement.

3. the comparison analysis of the proposed study with existing needs to be mentioned in both tabular and contexts.

Thank you. This study is the first of its kind to examine the efficiency of XLA for numerical models. Some existing studies examined the efficiency of XLA for machine-learning models and linear algebrea calculations, and these studies are referenced in the introduction section.

4. the pseudo code of the proposed methodology should be provided.

The codes used to reproduce the results of the current study are uploaded onto the GitHub repository and are publicly available at <https://github.com/xuzhen-he/XLA_numerical_models> (DOI: 10.5281/zenodo.7523671)

Reviewer #2:

Dear Authors, the manuscript titled “Accelerated linear algebra compiler for computationally efficient numerical models: success and potential area of improvement” is interesting and contains important information for readers. I can recommend this paper for publication with no modifications needed

Regards

Thank you. The manuscript is revised minorly to accommodate the suggestion of another reviewer and the journal requirements.
